# Enhancement of Roll-to-Roll Gravure-Printed Cantilever Touch Sensors via a Transferring and Bonding Method

**DOI:** 10.3390/s25030629

**Published:** 2025-01-22

**Authors:** Sang Hoon Lee, Jae Hak Shin, Sangyoon Lee

**Affiliations:** 1 Department of Chemical and Biomolecular Engineering, University of California, Irvine, Irvine, CA 92697, USA; sanghol9@uci.edu; 2 Department of Mechanical Design and Production Engineering, Konkuk University, Seoul 05029, Republic of Korea; dftd93@konkuk.ac.kr; 3 Department of Mechanical and Aerospace Engineering, Konkuk University, Seoul 05029, Republic of Korea

**Keywords:** capacitive touch sensor, air gap, cantilever, roll to roll, gravure printing, transferring and bonding method

## Abstract

Sensor miniaturization offers significant advantages, including enhanced SoC integration efficiency, reduced cost, and lightweight design. While the roll-to-roll printed electronics fabrication process is advantageous for the mass production of sensors compared to the traditional MEMS technology, producing sensors that require air gap-based 3D structures remains challenging. This study proposes an integration of roll-to-roll gravure printing with a transferring and bonding method for touch sensor fabrication. Unlike previously reported methods for sacrificial layer removal, this approach prevents stiction issues, thus enabling sensor miniaturization and providing the flexibility to select materials that minimize sensitivity degradation during scaling. For the lower part of the sensor, Ag and BaSO_4_ were roll-to-roll gravure-printed on a flexible PET substrate to form the bottom electrode and dielectric layer, followed by BaSO_4_ spin coating on the sensor’s anchor area to form a spacer. For the upper part, a water-soluble PVP sacrificial layer was roll-to-roll gravure-printed on another flexible PET substrate, followed by spin coating Ag and SU-8 to form the top electrode and the structural layer, respectively. The sacrificial layer of the upper part was removed with water to delaminate the top electrode and structural layer from the substrate, then transferred and bonded onto the spacer of the lower part. Touch sensors of three different sizes were fabricated, and their performances were comparatively analyzed along with that of an epoxy resin-based sensor, demonstrating that our sensor attained miniaturization while achieving relatively high sensitivity.

## 1. Introduction

The miniaturization of sensors is accelerating due to their advantages, including space efficiency, enhanced integration with system-on-chip (SoC) designs, low cost, light weight, and applicability across a wide range of applications [1,2,3,4,5]. These sensors are typically fabricated using the microelectromechanical systems (MEMS) process and encompass various types, including touch/pressure sensors, accelerometers, gyroscopes, temperature sensors, and gas sensors [6,7,8]. However, achieving sensor miniaturization necessitates a careful consideration of challenges such as performance degradation, reproducibility, and the complexity of the manufacturing process [9,10,11,12,13].

**Figure 1:**
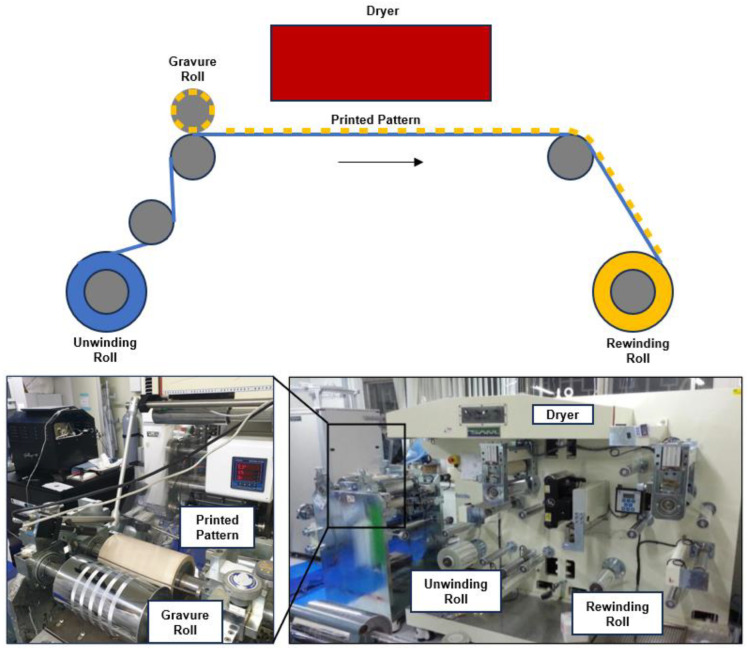
Schematic (**top**) and pictures (**bottom**) of roll-to-roll gravure printing process.

The roll-to-roll printed electronics fabrication process, which can potentially replace the MEMS process is being actively researched for the efficient production and manufacturing of flexible devices [14,15,16,17,18,19,20,21,22]. The roll-to-roll gravure printing process involves printing various types of functional solutions onto a moving flexible substrate to produce devices through large-scale production (Figure 1 and Supplementary Video S1) [23,24]. Despite its high efficiency, this method is limited to thin-film devices, making it challenging to fabricate sensors requiring three-dimensional (3D) structures traditionally produced using MEMS.

**Figure 2:**
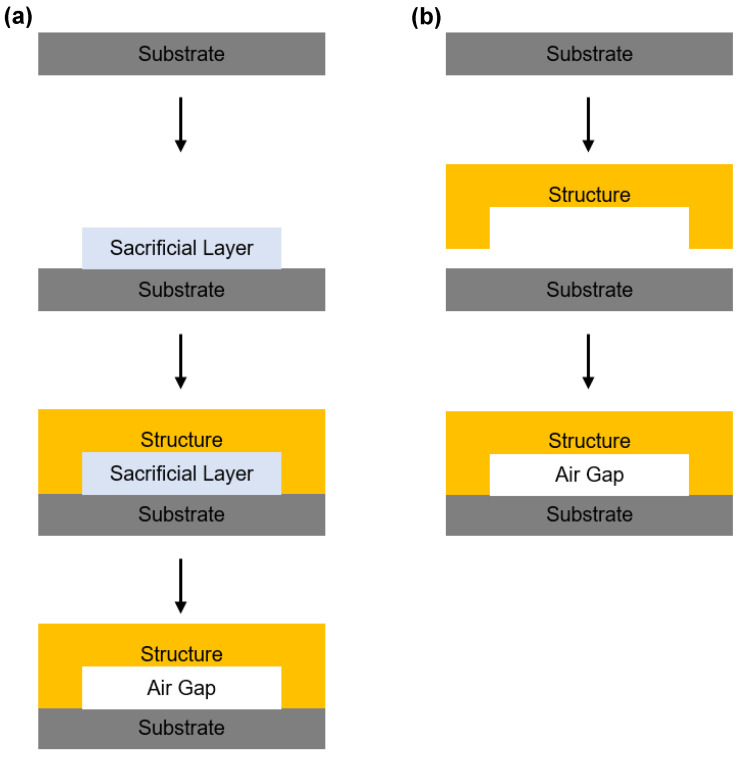
MEMS air gap structure fabrication processes. (**a**) Schematics showing sacrificial layer removal method and (**b**) transferring and bonding method.

In the MEMS process, 3D structures with air gaps are typically fabricated using methods such as sacrificial layer removal or transferring and bonding (Figure 2). Three-dimensionally structured air gap sensors surpass the widely researched polydimethylsiloxane (PDMS) sensors in terms of sensitivity, response speed, accuracy, reliability, and miniaturization potential [15,25,26]. In our previous work, we employed the sacrificial layer removal method (Figure 2a) [15,27,28,29,30]. Several three-dimensionally structured touch sensors, accelerometers, and actuators were fabricated and evaluated [27,28,29,30]. To create the air gap, sacrificial layers made of water-soluble polyvinyl alcohol (PVA) or polyvinylpyrrolidone (PVP) were printed or coated, dried, and subsequently removed [27,28,29,30]. For the structural layer of the sensors, we employed a relatively rigid epoxy resin to support the air gap even after the sacrificial layer removal [15,28,29,30]. The resulting air gap touch sensors demonstrated a 22-fold improvement in sensitivity compared to PDMS-based touch sensors under identical conditions [28]. Additionally, we successfully fabricated accelerometers, which were previously considered unachievable using roll-to-roll printed electronics [14,29]. However, similar to MEMS processes, this sacrificial layer removal method is prone to stiction issues, posing challenges to sensor miniaturization [31,32,33,34]. Consequently, the reported sensors had relatively large areas with dimensions in the centimeter range [15,16,28,29,30].

In this study, we employed a transferring and bonding method to fabricate touch sensors and address the limitations of miniaturization (Figure 2b). This method involves separately printing and coating the sensor layers on two different substrates and then integrating them, thereby mitigating stiction issues [35,36,37]. This stiction-free advantage broadens the selection of the structural layer materials [35,36,37]. We selected SU-8, which is commonly used as a structural layer material [38,39,40]. This material is easy to print or use as a coat in ultra-thin layers on the scale of a few nanometers, which helps to enhance sensitivity [38,39,40]. Additionally, its relatively high mechanical strength and stiffness make it ideal for maintaining the air gap structure [38,39,40]. Moreover, the transferring and bonding method enables a precise control over the air gap thickness by adjusting the thickness of a spacer [41,42]. We designed a spacer’s thickness of 120 µm. Sensors fabricated using this approach achieved a 7-fold reduction in area compared to previously reported touch sensors while significantly improving their sensitivity [28]. By adopting the roll-to-roll gravure printing process and optimizing the parameters, we achieved high productivity and efficiency in sensor fabrication (see Section 3.1).

We fabricated five SU-8-based large-area sensor samples, medium-area sensor samples, and small-area sensor samples. We additionally fabricated five epoxy resin-based large-area sensor samples as a control. First, to evaluate the performance of the sensor based on the material used for the structural layer, we measured, compared, and analyzed sensitivity and hysteresis characteristics of the SU-8-based and epoxy resin-based large-area sensor samples (see Section 3.2.1). Subsequently, to understand the impact of sensor size on its characteristics, we measured, analyzed, and compared the sensitivity, hysteresis, and response times of three differently sized sensor samples made of SU-8 (see Section 3.2.2). Finally, the SU-8-based miniaturized small-area sensor samples were compared with the epoxy resin-based large-area sensor samples in terms of size, sensitivity, and hysteresis characteristics, demonstrating significant improvements (see Section 3.2.3).

## 2. Materials and Methods

**Figure 3:**
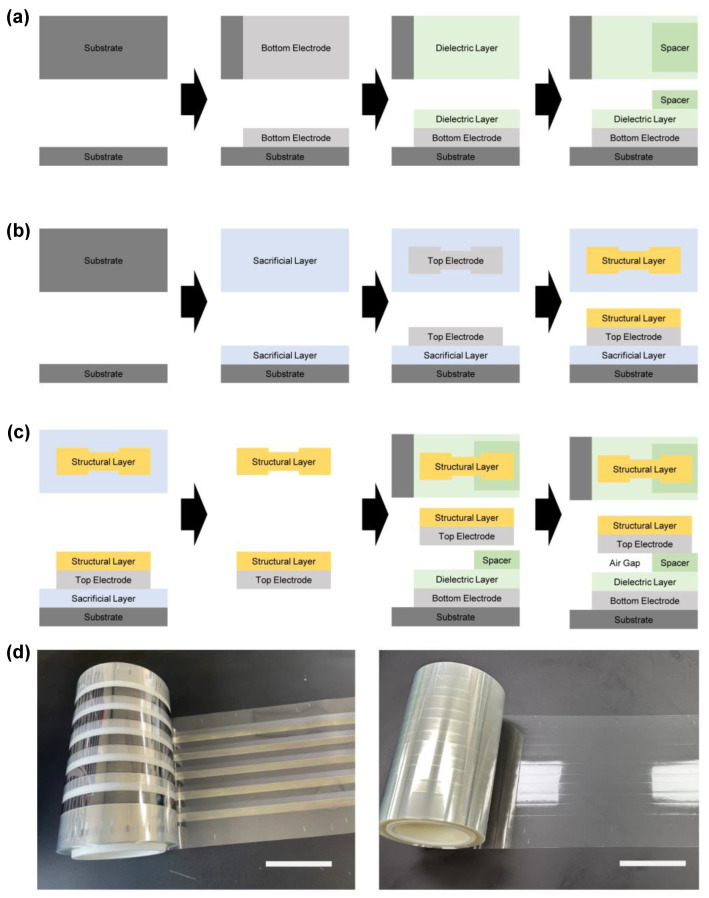
Fabrication of touch sensor samples. (**a**) Schematics showing fabrication sequence of lower part; (**b**) schematics showing fabrication sequence of upper part; (**c**) schematics showing delamination, transferring, and bonding of upper part; (**d**) roll-to-roll gravure-printed top electrode and dielectric layer (**left**) and roll-to-roll gravure-printed sacrificial layer (**right**). The scale bars are 100 mm.

For the fabrication of the touch sensor, the lower part, which includes the bottom electrode, dielectric layer, and spacer, and the upper part, which includes the sacrificial layer, top electrode, and structural layer, were roll-to-roll gravure-printed or spin-coated following the procedures shown in Figure 3. The patterns, used for the spin coating processes, were designed using AutoCAD software (v.2024), and sticky polyimide (PI) masks with these patterns were cut and fabricated using a Craft ROBO cutter (Graphtec, Tokyo, Japan). First, silver (Ag) ink (InkTec, Ansan, Republic of Korea, TEC-CO-021) was roll-to-roll gravure-printed on a flexible poly(ethylene terephthalate) (PET) substrate (SKC SH34) at a speed of 11 m/min, and the printed electrode was dried in an infrared oven installed in the roll-to-roll gravure printer at 250 °C for 26 s to form the bottom electrode. To protect the bottom electrode from contact with the top electrode, barium sulfate (BaSO_4_) ink (Toyobo, Osaka, Japan, SOC2808) was roll-to-roll gravure-printed at a speed of 3 m/min and dried in the oven at 180 °C for 1 min to form the dielectric layer. To achieve a sufficient air gap height of 120 µm, a spacer was formed by spin coating BaSO_4_ at 4000 rpm for 1 min onto the anchor area of the touch sensor sample, followed by drying on a hot plate at 150 °C for 30 min.

For the fabrication of the upper part, a new flexible PET substrate (SKC, Seoul, Republic of Korea, SH34) was prepared. A solution containing 10 wt % PVP powder (Comscience, Gwangju, Republic of Korea) dissolved in water was roll-to-roll gravure-printed at a speed of 5 m/min and dried in the infrared oven at 70 °C for 15 s to form the sacrificial layer. The sacrificial layer facilitates the delamination of the upper part’s components (i.e., the top electrode and structural layer) from the substrate. A stretchable Ag ink (Toyobo, SSP2801) was selected for the top electrode to withstand repeated touches without cracking. The Ag ink was spin-coated at 7000 rpm for 1 min and dried on a hot plate at 150 °C for 20 min. To enhance the durability of the top electrode, SU-8 was spin-coated at 4000 rpm for 1 min, soft-baked in a hot-air oven at 70 °C for 1 min, exposed to 365 nm UV light for 30 s, and hard-baked on a hot plate at 120 °C for 20 min to form the structural layer. For the control samples, epoxy resin (Alteco, Osaka, Japan, F-301) was spin-coated at 7000 rpm for 1 min and dried on a hot plate at 150 °C for 20 min to form the structural layer. Finally, the upper part was immersed in distilled water to remove the sacrificial layer and delaminate the components from the substrate. The delaminated freestanding components (i.e., the top electrode and structural layer) were attached to a PDMS mold (Dow Corning, Midland, TX, USA, Sylgard 184), transferred onto the spacer of the lower part, and bonded under pressure on a hot plate at 120 °C for 30 min.

The materials, processes, and drying conditions used are summarized in Table 1. The fabrication process described above was used to fabricate four different types of touch sensors (Table 2). The three differently sized touch sensors were designed to maintain consistent aspect ratios among the samples. Table 2 provides information on the area of the four different types of sensors, cantilever beam lengths, structural layer materials, and spacer layer thickness (which significantly affects the air gap thickness). The air gap thickness of the different types of sensor samples was measured using a field-emission scanning electron microscope (FE-SEM; Hitachi, Tokyo, Japan, SU8010). Relationships between force and capacitance, hysteresis curves, and the response times of the sensor samples were measured using an integrated system comprising a push–pull gauge (Mark-10, New York, NY, USA, Series 5), a parameter analyzer (Tektronix, Beaverton, OR, USA, Keithley 4200-SCS), and a probe station (MS Tech, Hwasung, Republic of Korea, M7VC).

## 3. Results

### 3.1. Optimization of the Roll-to-Roll Gravure Printing Process

The roll-to-roll process can be optimized to enhance productivity and printability [14,15,21]. In this study, optimization was primarily aimed at enhancing productivity. For the roll-to-roll gravure printing of the bottom electrode and dielectric layer, speeds ranging from 3 m/min to 15 m/min were considered. Through repeated experiments, it was confirmed that Ag coating at 11 m/min and BaSO_4_ printing at 3 m/min met the printability requirements. These speeds represent a 22-fold and 6-fold improvement, respectively, compared to the speed of 0.5 m/min used in previously published studies [28]. For the spacer, the coating speed required to achieve a thickness of 120 µm was determined to be 4000 rpm, based on tests conducted at speeds ranging from 3000 rpm to 7000 rpm for 1 min. For the sacrificial layer, speeds from 3 m/min to 15 m/min were considered, and it was confirmed through repeated experiments that printing PVP at a speed of 3 m/min satisfied the printability requirements. This represents a 10-fold improvement over the speed of 0.5 m/min used in previously published studies [28]. For the top electrode and structural layer, the spin coating speeds were selected to obtain the thinnest possible thickness while satisfying the coatability requirements.

**Figure 4:**
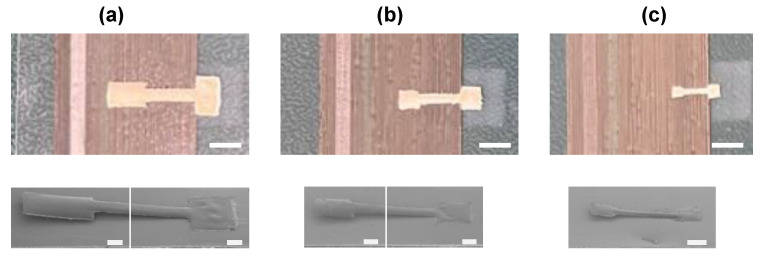
Digital camera images and SEM images of the roll-to-roll-fabricated touch sensor samples. (**a**) A digital camera image and an SEM image of the SU-8-based large-area touch sensor sample; (**b**) a digital camera image and an SEM image of the SU-8-based medium-area touch sensor sample; (**c**) a digital camera image and an SEM image of the SU-8-based small-area touch sensor sample. The scale bars on the digital camera images area 3 mm and on the SEM images are 500 µm.

The structure of the touch sensors fabricated using the optimized process parameters was evaluated using a digital camera and an FE-SEM (Figure 4). The areas of the touch sensors were 10, 4.95, and 1.44 mm^2^, with the smallest sensor sample being more than seven times smaller than the previously reported 10 mm^2^ touch sensor (Figure 4). The air gap height was 120 µm analogous to the one reported previously (Figure 4) [28]. No cracks or structural collapses were found in the touch sensor samples (Figure 4).

### 3.2. Characterization of Roll-to-Roll-Printed Touch Sensor Samples

**Figure 5:**
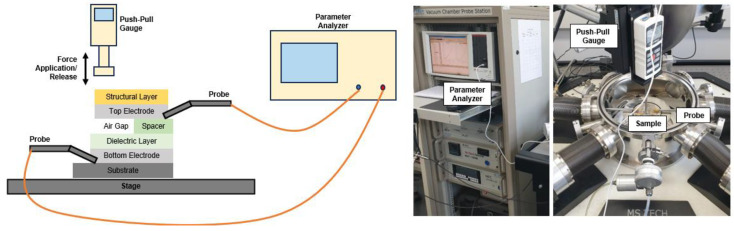
Schematics and pictures showing the characterization of roll-to-roll gravure-printed touch sensor samples.

The characteristics of the touch sensor samples were evaluated as shown in Figure 5. The sensor samples were first mounted on the stage of the probe station, and two probes in the probe station were put in contact with the bottom and top electrodes. Then, a push–pull gauge mounted on a motorized stand was moved up or down to apply force to the sensor samples. The force applied to the sensors via the push–pull gauge and the capacitance values obtained for the sensor samples were recorded in real time in the analyzer. The frequency of the parameter analyzer was 1 MHz.

The capacitance value can be calculated using Equation (1) [43,44,45]. As shown in the equation, the capacitance changes based on the *d* value, which varies with the force applied to or released from the sensor [43,44,45].

(1) $$C=\varepsilon_{0}\cdot \varepsilon_{r}\cdot \frac{A}{d}$$

Here, *C* represents the capacitance, *ε*_0_ represents the electric permittivity of vacuum, *ε_r_* represents the relative permittivity of the dielectric, *A* represents the area, and *d* represents the thickness of the dielectric [43,44,45].

The sensitivity was calculated using Equation (2) [45,46]. In this study, the push–pull gauge mounted on a motorized stand was used to apply force at a rate of 0.4 µm/s up to a maximum of 1.3 N to evaluate the sensitivity of the sensor samples. The capacitance values of the sensors were measured and recorded in real time during this process. The obtained data were substituted into Equations (2) to calculate the sensitivity.

(2) $$S=\frac{\Delta C/C_{0}}{\Delta P}$$

Here, *S* represents the sensitivity, Δ*P* represents the change in pressure (or force), and Δ*C/C*_0_ represents the rate of capacitance change [45,46].

In this study, we additionally evaluated the sensor samples’ hysteresis characteristics. To obtain the hysteresis curves for the sensor samples, the push–pull gauge mounted on the motorized stand applied force at a rate of 0.4 µm/s up to a maximum of 1.3 N and then released the force back to 0 N at the same rate. The capacitance values of the sensors were measured and recorded in real time during this process. The obtained data were substituted into Equations (3) and (4) to calculate the maximum hysteresis value [47,48].

(3) $$u_{h}=\left|{\left(y\right)}_{upscale}-{\left(y\right)}_{downscale}\right|$$

(4) $${\%u}_{hmax}=\frac{u_{hmax}}{r_{0}}\cdot 100=\frac{u_{hmax}}{y_{max}-y_{min}}\cdot 100$$

Here, *u_h_* represents the hysteresis error, (*y*)*_upscale_* represents the capacitance when force is applied, and (*y*)*_downscale_* represents the capacitance when force is released. *u_hmax_* represents the maximum hysteresis error, and *r*_0_ represents the full-scale output range (i.e., *y_max_* – *y_min_*) [47,48].

Finally, to measure the response time, the push–pull gauge mounted on the motorized stand applied force at a rate of 1.2 mm/s up to a maximum of 1.3 N, and then released the force back to 0 N at the same rate. This process was repeated twice. The capacitance values of the sensor were measured and recorded in real time during this process. The data obtained were substituted into Equation (5) to calculate the sensor’s response time.

(5) $$\tau =\frac{∆C}{\frac{dC}{dt}}$$

Here, *τ* represents the response time, Δ*C* represents the total capacitance change, and *dC*/*dt* represents the rate of capacitance change over time.

For each measurement, four types of sensor samples including epoxy resin-based large-area, SU-8-based large-area, SU-8-based medium-area, and SU-8-based small-area sensors were prepared. First, to understand the impact of the structural layer material on sensor performance, the sensitivity and hysteresis of the epoxy resin-based and SU-8-based large-area sensor samples were measured, analyzed, and compared (see Section 3.2.1). Second, to investigate the effect of the sensor area on its characteristics, the sensitivity, hysteresis, and response time of the SU-8-based small-area, medium-area, and large-area sensor samples were measured, analyzed, and compared (see Section 3.2.2). Lastly, the miniaturized SU-8-based small-area sensor samples were compared to the epoxy-based large-area sensor samples in terms of sensitivity and hysteresis to analyze the improvement in the miniaturized SU-8-based small-area sensor samples’ characteristics (see Section 3.2.3). For all performance evaluations, five sensor samples of each type were used.

#### 3.2.1. Effects of the Structural Layer Materials on the Touch Sensors’ Performance

The material of the structural layer in the cantilever beam of cantilever-structured sensors is a critical factor in determining sensitivity, reliability, and suitability for specific applications [49,50,51]. Common materials for the structural layer include silicon, silicon nitride, metals such as gold (Au), chromium (Cr), and titanium (Ti), composite materials like carbon nanotubes and graphene, and polymers such as SU-8, epoxy resin, and PDMS [52,53,54,55]. These materials are selected based on their mechanical properties, thermal characteristics, and chemical stability to meet the requirements of the intended application [52,53,54,55]. For example, cantilever sensors with polymer beams are often used in bio-, chemical, or wearable sensors due to their high flexibility, sensitivity, and biocompatibility [56,57].

In our previous study, the use of structural layer materials other than epoxy resin was challenging due to stiction issues arising from the sacrificial layer removal process [28]. The transferring and bonding method addresses these stiction issues, enabling the adoption of all the aforementioned materials depending on the application. In this study, SU-8, a commonly used structural material, was adopted, and the performance of the obtained sensors was compared to that of sensors with an epoxy resin structural layer with the same area and configuration.

Five SU-8-based large-area touch sensor samples with an area of 10 mm^2^ and five epoxy-based large-area touch sensor samples with the same area were prepared, and their sensitivity and hysteresis were measured, calculated, analyzed, and compared following the procedures written in Section 3.2.

**Figure 6:**
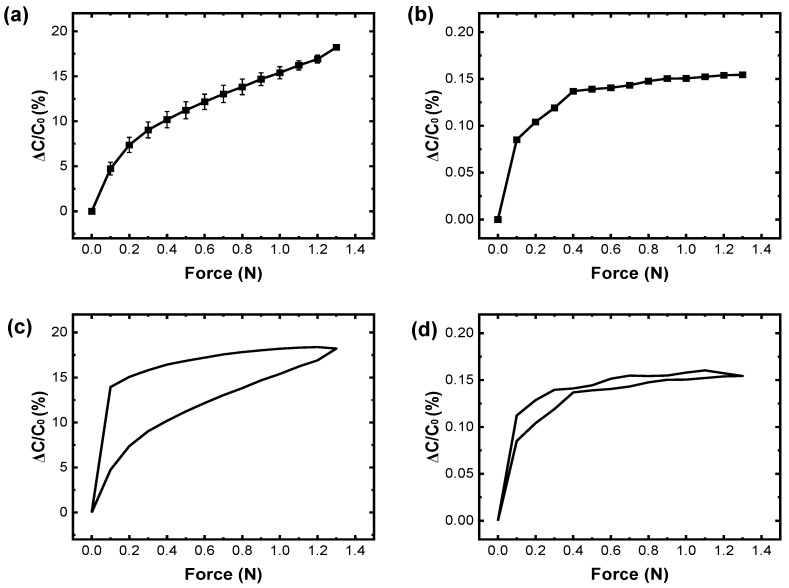
Relationship between force and capacitance changes for SU-8-based large-area touch sensor samples and epoxy resin-based large-area touch sensor samples. (**a**) A plot showing the relationship between force and capacitance changes for the SU-8-based large-area touch sensor samples; (**b**) a plot showing the relationship between force and capacitance changes for the epoxy resin-based large-area touch sensor samples; (**c**) a plot showing the hysteresis curve for the SU-8-based large-area touch sensor samples; (**d**) a plot showing the hysteresis curve for the epoxy resin-based large-area touch sensor samples.

First, to calculate the sensitivities of the sensor samples, capacitance changes according to the applied force for the SU-8-based and epoxy resin-based large-area sensor samples were obtained as shown in Figure 6a,b. A plot of the relationship between capacitance changes and applied force obtained for the SU-8-based large-area touch sensor samples is shown in Figure 6a. The average capacitance changes of the SU-8-based large-area touch sensor samples increased linearly up to 0.4 N and saturated beyond 0.4 N (Figure 6a). The average sensitivity of the SU-8-based large-area touch sensor samples was 24.67%/N in the linear region and 8.50%/N in the saturation region (Figure 6a and Table 3). The average capacitance changes of the epoxy-based large-area touch sensor samples increased linearly up to 0.4 N and saturated beyond 0.4 N (Figure 6b). The average sensitivity of the epoxy-based large-area touch sensor samples was 0.31%/N in the linear region and 0.02%/N in the saturation region (Figure 6b and Table 3). The average sensitivity of the SU-8-based large-area touch sensor samples was 80 times higher than that of the epoxy-based touch sensor samples in the linear region and 425 times higher in the saturation region.

Next, hysteresis curves were obtained for the SU-8-based and epoxy resin-based large-area sensor samples (Figure 6c,d). The average capacitance changes for the SU-8-based large-area touch sensor samples increased as force was applied up to 1.3 N and decreased as the force was released (Figure 6c). The maximum hysteresis error was 51%, observed at 0.1 N (Figure 6c and Table 3). Similarly, for the epoxy-based large-area touch sensor samples, the average capacitance change increased up to 1.3 N and decreased when the force was released (Figure 6d). The maximum hysteresis error was 18%, observed at 0.1 N (Figure 6d and Table 3). The maximum hysteresis error for the SU-8-based large-area touch sensor samples was almost three times higher than that for the epoxy-based large-area touch sensor samples.

The comparative analysis results demonstrated that the SU-8-based touch sensors exhibited significantly superior sensitivity compared to the epoxy-based touch sensors. This significant improvement is attributed to the relatively high stiffness and low dielectric constant of SU-8. For hysteresis, the SU-8-based touch sensors exhibited a higher maximum hysteresis error than the epoxy-based touch sensors. This difference is likely to be attributed to the relatively high stiffness and low viscoelastic properties of SU-8. These findings show that the transferring and bonding method, applicable to components printed using roll-to-roll processes, resolves stiction issues and broadens the selection of structural layer materials. This potential of the transferring and bonding method suggests that flexible sensors with either higher sensitivity or reduced hysteresis errors can be manufactured via roll-to-roll processes by selecting polymers with lower stiffness and higher elasticity.

#### 3.2.2. Effect of the Touch Sensor Area on Touch Sensors’ Performance

The area for capacitive touch sensors with cantilever structures is determined by the intended application [58,59]. Relatively large-area touch sensors are typically used in applications that require stable force or pressure detection over a wide range, whereas smaller area touch sensors are preferred for applications requiring the detection of a stable force or pressure [60,61]. Small-area touch sensors are advantageous for detecting delicate biological or chemical signals, which makes them suitable for applications in bio-, chemical, and wearable sensors [58,59,60,61].

In our previous study, the removal of the sacrificial layer made it challenging to fabricate touch sensors smaller than 10 mm^2^ due to stiction issues [28]. On the other hand, the transferring and bonding method resolves these stiction issues, enabling the fabrication of sensors of any size depending on the application [35,36,37]. In this study, SU-8, verified in Section 3.2.1 as a structural layer material, was used to fabricate sensor samples of three different sizes, i.e., 1.44 (small-area sensor), 4.95 (medium-area sensor), and 10 mm^2^ (large-area sensor), and their performances were compared.

Three types of SU-8-based touch sensors were designed with the same aspect ratio, and five samples were prepared for each type. The sensitivity, hysteresis, and response time of these samples were measured, calculated, and analyzed following the procedures described in Section 3.2.

**Figure 7:**
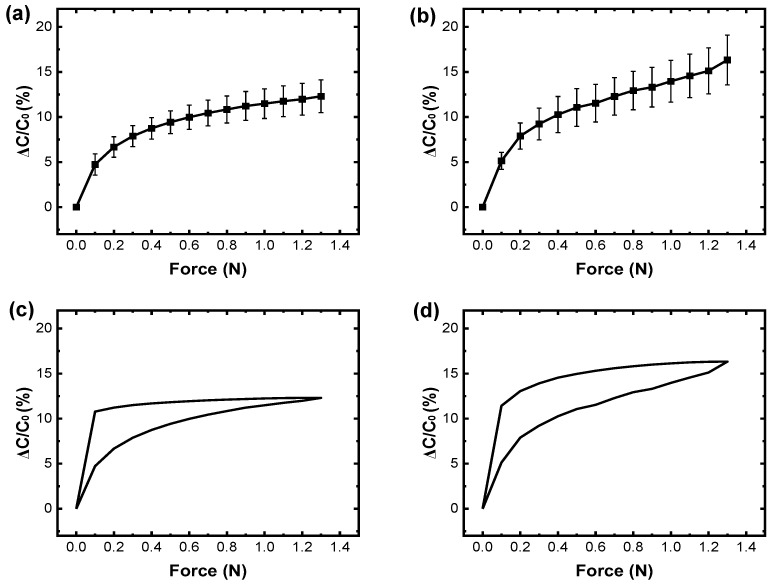
Relationship between force and capacitance changes for the SU-8-based small-area touch sensor samples. (**a**) A plot showing the relationship between force and capacitance changes for the SU-8-based small-area touch sensor samples; (**b**) a plot showing the relationship between force and capacitance changes for the epoxy resin-based medium-area touch sensor samples; (**c**) a plot showing the hysteresis curve for the SU-8-based small-area touch sensor samples; (**d**) a plot showing the hysteresis curve for the epoxy resin-based medium-area touch sensor samples.

First, to calculate the sensitivity of the sensor samples, capacitance changes according to the applied force for the SU-8-based small-area, medium-area, and large-area sensor samples were obtained as shown in Figure 7a, Figure 7b, and Figure 6a, respectively. The average capacitance changes of the small-area touch sensor samples increased linearly up to 0.4 N and saturated beyond 0.4 N (Figure 7a). The average sensitivity of the small-area touch sensor samples was 20.64%/N in the linear region and 3.77%/N in the saturation region (Figure 7a and Table 4). Similarly, the medium-area touch sensor samples showed linear capacitance increases up to 0.4 N, followed by saturation beyond 0.4 N (Figure 7b). The average sensitivity of the medium-area touch sensor samples was 24.62%/N in the linear region and 6.27%/N in the saturation region (Figure 7b and Table 4). The average sensitivity in the linear region obtained for the small-area sensor samples was 4.03%/N lower than the average sensitivity obtained for the large-area sensor samples and 3.98%/N lower than the average sensitivity obtained for the medium-area sensor samples (Figure 6a and Figure 7a,b). And the average sensitivity in the saturation region obtained for the small-area sensor samples was 4.73%/N lower than the average sensitivity obtained for the large-area sensor samples and 2.50%/N lower than the average sensitivity obtained for the medium-area sensor samples (Figure 6a and Figure 7a,b). In contrast, the average sensitivities in both the linear and the saturation regions obtained for the medium-area and large-area touch sensor samples were similar (Figure 6a and Figure 7b, and Table 4). The lower sensitivity of the small-area sensor samples may be caused by the dependence of capacitance on the sensor’s area, as shown in Equation (1).

Next, the hysteresis curves obtained for the SU-8-based small-area, medium-area, and large-area sensor samples are shown in Figure 7c, Figure 7d and Figure 6c, respectively. The average capacitance changes for the small-area touch sensor samples increased as force was applied (up to 1.3 N) and decreased as the force was released (Figure 7c). The maximum hysteresis error was 49%, found at 0.1 N (Figure 7c and Table 4). Similarly, the medium-area touch sensor samples exhibited capacitance increases and decreases under the same conditions, with a maximum hysteresis error of 39%, found at 0.1 N (Figure 7d and Table 4). These relatively high maximum hysteresis errors were found for all these touch sensor samples with small area, medium area, and large area. These high errors were attributed to the relatively high stiffness and low viscoelastic properties of SU-8.

**Figure 8:**
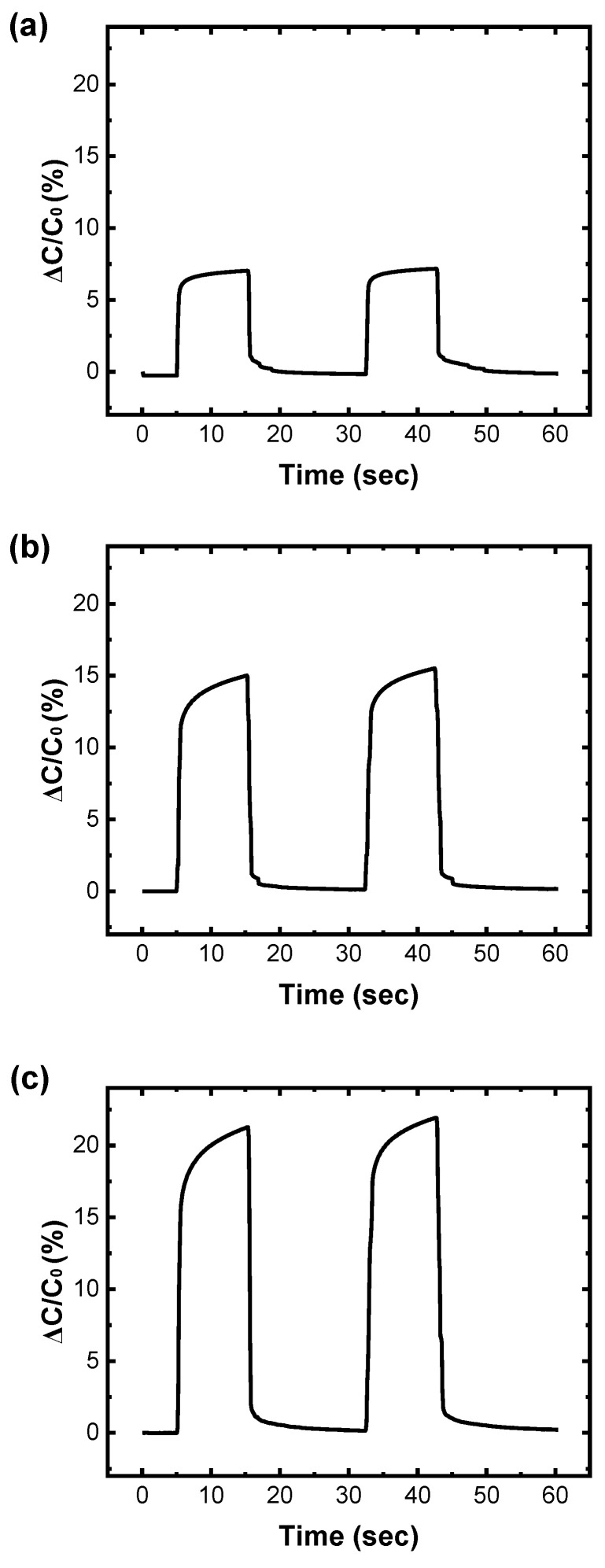
Switching performances of the SU-8-based touch sensor samples. (**a**) A plot showing the switching performances of the SU-8-based large-area touch sensor samples; (**b**) a plot showing the switching performances of the SU-8-based medium-area touch sensor samples; (**c**) a plot showing the switching performances of the SU-8-based small-area touch sensor samples.

Finally, the response times were obtained for the SU-8-based small-area, medium-area, and large-area sensor samples from the switching plots shown in Figure 8a, 8b, and 8c, respectively. The average capacitance changes for the small-area touch sensor samples increased under a force of 1.3 N held for 10 s and decreased when the force was released (Figure 8a). The average response time of the small-area touch sensor samples was 280 ms (Figure 8a and Table 4). Similarly, the medium-area touch sensor samples exhibited an average response time of 280 ms (Figure 8b and Table 4). The large-area touch sensor samples followed the same trend, with an average response time of 310 ms (Figure 8b and Table 4). The difference in the area of our sensor samples did not appear to significantly affect the response speed (Figure 8 and Table 4).

Here, we evaluated the sensitivity, hysteresis, and response time of the three different types of sensor samples with different sizes. While the sensitivity of the small-area sensor sample was slightly lower than that of the other sensors of different sizes, no significant performance degradation related to the size was observed overall. These results demonstrate that our findings are not only significant compared to those of our previous studies but also notable when compared to those for recently reported PDMS-based sensors, despite differences in size and configuration [28,30,62,63,64]. For example, sensitivity was reported as 1.57%/N, hysteresis error as up to 21.04%, and response time as up to 0.38 seconds [62,63,64].

In this study, a manual transferring and bonding process using PDMS was employed, which limited the ability to fabricate ultra-miniature sensors. However, it is expected that the application of three-axis micro transfer equipment could enable the fabrication of ultra-miniature sensors, at the micro or nano scale, tailored for specific applications.

#### 3.2.3. Performance Analysis of the Miniaturized Touch Sensor Samples

In this section, we ultimately analyze, based on the data obtained in Section 3.2.1 and Section 3.2.2, how the miniaturized small-area sensor is improved in terms of size, sensitivity, hysteresis, and response time compared to epoxy-based large-area sensors, which are analogous to the sensors previously reported.

**Figure 9:**
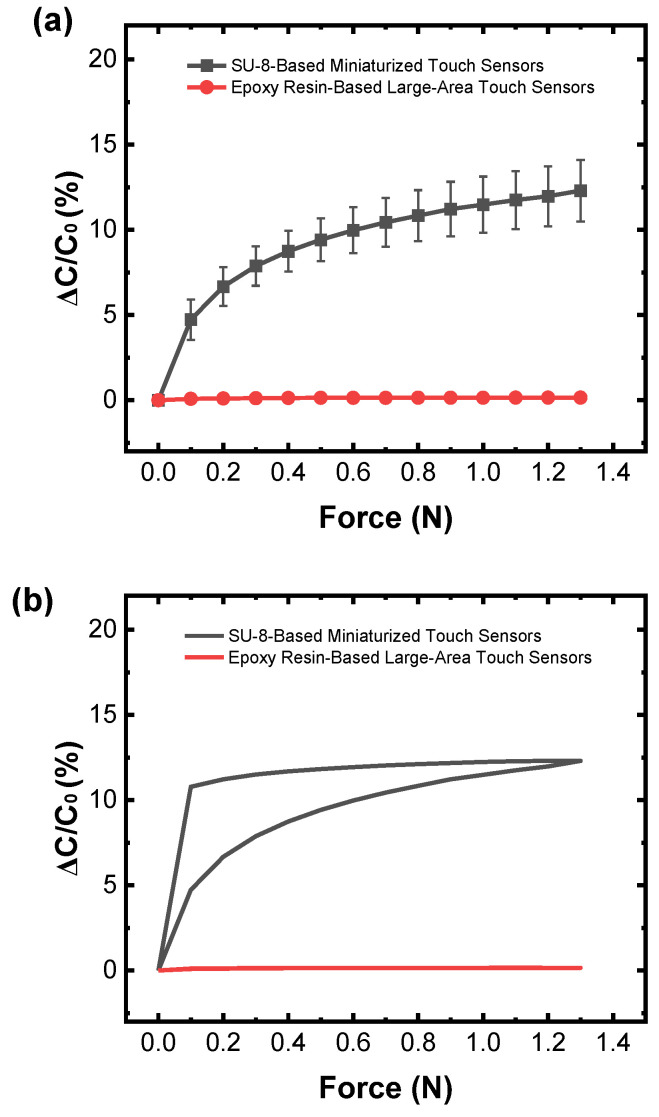
Relationship between force and capacitance changes for the SU-8-based small-area touch sensor samples and epoxy resin-based large-area touch sensor samples. (**a**) A plot showing the relationship between force and capacitance changes for both SU-8-based small-area touch sensor samples and epoxy resin-based large-area touch sensor samples; (**b**) a plot showing the hysteresis curves for both SU-8-based small-area touch sensor samples and epoxy resin-based large-area touch sensor samples.

First, for the comparative analysis of sensitivity, the relationship between capacitance changes and force is shown in Figure 9a. In the linear region, the average sensitivity of the SU-8-based small-area touch sensor samples was 67 times higher than that of the epoxy-based large-area touch sensor samples. In the saturation region, the average sensitivity of the SU-8-based small-area touch sensor samples was 189 times higher than that of the epoxy-based large-area touch sensor samples.

Next, for the comparative analysis of hysteresis, the hysteresis curves are shown in Figure 9b. The maximum hysteresis error for the SU-8-based small-area touch sensor samples was 31% higher than that for the large-area epoxy-based touch sensor samples.

Based on the comparative analysis, the SU-8-based touch sensors exhibited significantly higher sensitivity compared to the epoxy-based touch sensors, though the issue of high hysteresis remains a challenge to address. These results demonstrate that the transfer and bonding method applicable to roll-to-roll printed components effectively mitigates stiction issues, thereby expanding the range of materials that can be used in structural layers. The potential of this transfer and bonding method suggests that by selecting low-stiffness, highly elastic polymers, flexible sensors with higher sensitivity or lower hysteresis errors can be fabricated using roll-to-roll processes.

## 4. Conclusions

This study successfully demonstrated the integration of roll-to-roll gravure printing with a transfer and bonding method to fabricate miniaturized touch sensors with air gap-based 3D structures. By addressing stiction issues, the proposed approach enables sensor miniaturization while maintaining high sensitivity and scalability.

The optimized roll-to-roll printing process achieved significant productivity enhancements, with up to 22-fold faster production rates for key components compared to previously reported methods. Using this process, SU-8-based touch sensors of three different sizes were fabricated and systematically evaluated in terms of sensitivity, hysteresis, and response time. The results showed that the sensitivity of the SU-8-based small-area sensor samples was 67 times higher compared to that of the epoxy-based large-area sensor samples, which were used as a control and are structurally analogous to previously reported sensors [28]. This significant improvement highlights the advantage of SU-8 as a structural material to achieve higher sensitivity. Despite these significant improvements in sensitivity, the SU-8 sensors exhibited higher hysteresis errors. However, these limitations could potentially be addressed through further material and design optimization, paving the way for improved performance in future iterations of the sensors. Additionally, the response time remained consistent across all sizes, indicating minimal performance degradation due to miniaturization.

This study highlights the versatility and scalability of the proposed fabrication method, which not only resolves stiction issues but also broadens the choice of structural materials for touch sensors, enabling the development of high-performance, flexible devices. Importantly, the results demonstrate the potential for manufacturing ultra-miniature sensors through the employment of advanced three-axis micro-transfer equipment. This approach opens new possibilities for fabricating sensors at the micro- or nanoscale, tailored to applications requiring exceptional precision and sensitivity, such as wearable electronics and biomedical devices.

Future work will focus on further material optimization, particularly through the use of low-stiffness and high-elasticity polymers, to reduce hysteresis and enhance device performance. Additionally, employing automated micro-transfer equipment could enable the fabrication of ultra-miniature sensors at the micro- or nanoscale, unlocking new possibilities for applications in wearable electronics, biomedical devices, and IoT systems.

## Figures and Tables

**Table 1:** Materials and fabrication process conditions for layers of the touch sensor.

	Layer	Material	Process	Printing Condition	Drying Condition
Lower	Bottom Electrode	Ag	Roll-to-Roll Gravure Printing	11 m/min	250 °C for 26 s
	Dielectric	BaSO_4_	Roll-to-Roll Gravure Printing	3 m/min	180 °C for 1 min
	Spacer	BaSO_4_	Spin Coating	4000 rpm for 1 min	150 °C for 30 min
Upper	Sacrificial	PVP	Roll-to-Roll Gravure Printing	5 m/min	70 °C for 15 s
	Top Electrode	Stretchable Ag	Spin Coating	7000 rpm for 1 min	150 °C for 20 min
	Structural	SU-8/Epoxy	Spin Coating	4000 rpm for 1 min/ 7000 rpm for 1 min/	UV Curing (365 nm)/ 150 °C for 20 min

**Table 2:** Information about the four different types of sensor samples.

Type	Total Area	Cantilever Beam Length	Sacrificial Layer Material	Spacer Thickness
SU-8-Based Small-Area Touch Sensor	1.44 mm^2^	3 mm	SU-8	120 µm
SU-8-Based Medium-Area Touch Sensor	4.95 mm^2^	5 mm	SU-8	120 µm
SU-8-Based Large-Area Touch Sensor	10 mm^2^	7 mm	SU-8	120 µm
Epoxy Resin-Based Large-Area Touch Sensor	10 mm^2^	7 mm	Epoxy Resin	120 µm

**Table 3:** Characteristics of the SU-8-based and epoxy resin-based large-area touch sensor samples.

Type	Average Sensitivity in Linear Region	Average Sensitivity in Saturation Region	Maximum Hysteresis Error
SU-8-Based Large-Area Touch Sensor	24.67%/N	8.50%/N	51%
Epoxy Resin-Based Large-Area Touch Sensor	0.31%/N	0.02%/N	18%

**Table 4:** Characteristics of the SU-8-based small-area, medium-area, and large-area touch sensor samples.

Type	Average Sensitivity in Linear Region	Average Sensitivity in Saturation Region	Maximum Hysteresis Error	Response Time
SU-8-Based Small-Area Touch Sensor	20.64%/N	3.77%/N	49%	280 ms
SU-8-Based Medium-Area Touch Sensor	24.62%/N	6.27%/N	39%	280 ms
SU-8-Based Large-Area Touch Sensor	24.67%/N	8.50%/N	51%	310 ms

## Data Availability

The roll-to-roll gravure printing process is shown in Supplementary Videos.

## Supplementary Materials

The following supporting information can be downloaded at: https://www.mdpi.com/article/10.3390/s25030629/s1, Supplementary Video S1: Roll-to-roll printing process.
